# Irrigation-Induced Changes in Chemical Composition and Quality of Seeds of Yellow Lupine (*Lupinus luteus* L.)

**DOI:** 10.3390/ijms20225521

**Published:** 2019-11-06

**Authors:** Justyna T. Polit, Iwona Ciereszko, Alina T. Dubis, Joanna Leśniewska, Anna Basa, Konrad Krajewski, Aneta Żabka, Marharyta Audzei, Łukasz Sobiech, Agnieszka Faligowska, Grzegorz Skrzypczak, Janusz Maszewski

**Affiliations:** 1 Department of Cytophysiology, Institute of Experimental Biology, Faculty of Biology and Environmental Protection, University of Lodz, Pomorska 141/143, 90-236 Lodz, Poland; konrad.winnicki@biol.uni.lodz.pl (K.K.); aneta.zabka@biol.uni.lodz.pl (A.Ż.); avdejka.margo@gmail.com (M.A.); janusz.maszewski@biol.uni.lodz.pl (J.M.); 2 Department of Plant Biology and Ecology, Faculty of Biology, University of Bialystok, Ciołkowskiego1J, 15-245 Bialystok, Poland; icier@uwb.edu.pl (I.C.); joanles@uwb.edu.pl (J.L.); 3 Department of Organic Chemistry, Faculty of Chemistry, University of Bialystok, Ciołkowskiego1K, 15-245 Bialystok, Poland; alina@uwb.edu.pl; 4 Department of Physical Chemistry, Faculty of Chemistry, University of Bialystok, Ciołkowskiego 1K, 15-245 Białystok, Poland; abasa@uwb.edu.pl; 5 Agronomy Department, Poznań University of Life Sciences, Dojazd 11, 60-632 Poznań, Poland; lukasz.sobiech@up.poznan.pl (Ł.S.); agnieszka.faligowska@up.poznan.pl (A.F.); grzegorz.skrzypczak@up.poznan.pl (G.S.)

**Keywords:** endoreplication, FTIR, germination, mitotic activity, SEM-EDS, storage proteins

## Abstract

The quality and amount of yellow lupine yield depend on water availability. Water scarcity negatively affects germination, flowering, and pod formation, and thus introduction of an artificial irrigation system is needed. The aim of this study was to evaluate the influence of irrigation on the quality of yellow lupine seeds. Raining was applied with a semi-solid device with sprinklers during periods of greatest water demand. It was shown that watered plants produced seeds of lesser quality, having smaller size and weight. To find out why seeds of irrigated plants were of poor quality, interdisciplinary research at the cellular level was carried out. DNA cytophotometry evidenced the presence of nuclei with lower polyploidy in the apical zone of mature seeds. This may lead to formation of smaller cells and reduce depositing of storage materials. The electrophoretic and Fourier transform infrared spectroscopic analyses revealed differences in protein and cuticular wax profiles, while scanning electron microscopy and energy dispersive spectroscopy revealed, among various chemical elements, decreased calcium content in one of seed zones (near plumule). Seeds from irrigated plants showed slightly higher germination dynamics but growth rate of seedlings was slightly lower. The studies showed that irrigation of lupine affected seed features and their chemical composition, an ability to germination and seedlings growth.

## 1. Introduction

Yellow lupine (*Lupinus luteus* L.) is a legume crop which has tremendous economic potential and is of great importance both in sustainable agriculture, particularly in reclamation of marginal lands, and as a natural source of nitrogen thus it could be one of the main species cultivated for green fertilizer, seeds, food and feed. As a rotation crop, it reconstructs the soil after cereals, thus it plays a phytosanitary role. Perfectly developed pile root systems of lupine meliorate the soil, making its aeration and watering easier. In this way, it improves the water-air ratio and makes the damaged soil structure (resulting e.g., from cereal monoculture) more crumbly. As a result of symbiosis with papillary bacteria, lupine has the ability to bind indirectly free atmospheric nitrogen and, thus, to improve soil fertility. Its long roots take up ions of calcium, magnesium, potassium and phosphorus from deep layers of soil, inaccessible for other plant species. Thus, it increase the yield of follow-up crop, because the compounds stored in the tissues (macro- and micronutrients) return to the soil from crop residues (such as roots or straw), remaining after the harvest or when lupine plants are intended for plowing as a green fertilizer [1,2,3,4,5]. Yellow lupine contains a large amount of high quality proteins in its seeds and negligible amounts of harmful, bitter alkaloids. These proteins, due to the favorable amino acid composition, are of much higher quality than those derived from cereal grains. Therefore, the seeds are used as a protein source in the diets of livestock, and also as a component of food, especially functional food for people [1,4,5,6,7,8,9]. The lupine proteins have pharmaceutical qualities. They influence lipid and glucose metabolism as well as blood pressure. They may also affect inflammatory processes and changes in gut microbiome. This has a significant influence on the metabolism, nutrient absorption, and immune functions [4,10,11,12]. In addition, yellow lupine seeds are abundant in the Fe-rich ferritin and may be a safe way to increase dietary iron intake replacing traditional iron supplementation methods [13].

Yellow lupine is a species with the lowest soil requirements among other lupine plants, however, is characterized by a relatively long growing season until the seeds are produced. The atmospheric conditions prevailing at this time may both favor or impair the course of cultivation. During vegetation its yielding is unstable under unfavorable weather conditions, such as drought [5,9]. Due to this, it is not willingly grown despite its beneficial properties. Therefore, it is difficult to achieve such amounts of this high-protein crop seeds that could be competitive with soya, which now satisfies great part of nutritional needs, especially in Europe [4].

Thanks to the well-developed root system, plants of yellow lupine can cope with periodic water shortages, taking it from deeper soil layers, inaccessible for other herbaceous plants. However, long-lasting drought causes changes at the physiological and molecular level. Lack of water or its insufficient amount prolongs the flowering period. Water shortage activates stress responses and decreases the numbers of both flowers and developing pods, thus limiting lupine yield [5,9,14,15,16]. Drought inhibits the development of symbiotic bacteria from the *Rhizobium* group, and consequently decreases the total plant mass. In addition, water-deficit conditions can influence seed chemical composition, e.g., increasing alkaloid content in some sweet lupine varieties making them less attractive for farm animals [2,14,15,16,17,18]. Generally, shortage of water increases the production of reactive oxygen species in cells (which may cause damages in photosystems, especially PSII and in membranes of thylakoids), and decreases the rate of photosynthesis, due to low CO_2_ uptake, a lowered activity of photosynthetic enzymes and reduced chlorophyll content [17,19,20,21,22,23,24]. All changes in plant metabolism which ensure survival of unfavorable conditions limit crop yield [18,21,25]. Every reduction of agricultural productivity causes economic losses among farmers and increases food prices [26,27].

Complexity of plant response to water deficit makes genetic research that could lead to obtaining drought-resistant crops difficult and time-consuming [4,20,21,24]. Thus, different methods of irrigation are still the most common approach to reduce adverse effects of drought in agriculture. It was found that they increase crop productivity and seeds quality, [18,25,28,29,30,31,32,33]. However, contradictory results concerning the lupine are also known, and they indicate that irrigation might reduce seed vigor, germination capacity and germination energy, but increase a share of mold, rotting, and dead seeds [34,35]. It is therefore necessary to analyze whether irrigation is beneficial in all circumstances, even when plants are exposed only to mild water stress. If the seeds are to be used for consumption purposes, a specially high level of quality is desired, but if they are treated as a planting material, it is possible that mild drought will increase plant resistance to stress and the memory of stress will help to tolerate unfavorable conditions by the next generation of plants.

The aim of the current research was to investigate cytological, chemical and biochemical traits which may be responsible for quality of seeds from irrigated plants of yellow lupine. The obtained results indicate that the seeds harvested from the non-irrigated and irrigated plants differ in size and weight, endopolyploidy level of cotyledon cells, content of storage proteins, protein composition and in cuticular wax profiles, as well as they differ in the germination capacity and growth rate of embryonic roots.

## 2. Results

### 2.1. Seed Yield

Irrigation did not improve the seed yield of the yellow lupine. The amount of yield collected from the main stem as well as from branches of irrigated plants was comparable to that obtained from the control (non-irrigated) plants (Table 1). However, the seed yield was visually of inferior quality. For clarity, in the following sections of the work the seeds collected from the plants growing under natural conditions (without additional irrigation) are referred to as “control seeds” whereas the seeds from the plants subjected to irrigation are referred to as “irrigated seeds”.

### 2.2. Seed Morphology

**Figure 1:**
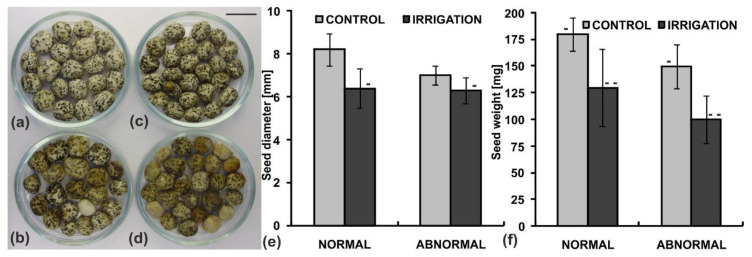
Seeds of yellow lupine collected from the control (not irrigated) and irrigated plants and sorted according to morphological features: (**a**) control—normal seeds, (**b**) control—abnormal seeds, (**c**) irrigated plants—normal seeds, (**d**) irrigated plants—abnormal seeds. Scale bar 10 mm, (**e**) seed size (diameter) measured along the long axis, (**f**) seed weight. Statistical significance between mean values of seed diameters and seed weights was assessed with the Mann–Whitney U test *(p <* 0.01) and Student’s t test (*p* < 0.01), respectively. Error bars represent standard deviation (SD). Minuses and double minuses indicate pairs of statistically insignificant results.

In both control and irrigated plants there were seeds of different morphology and quality. Therefore the control and the irrigated seeds were divided into normal (correct) and abnormal (incorrect) groups due to differences in their morphological state (Figure 1a–d).

The evaluation was based on seeds color, shape, estimated size and weight. Seeds from the first group were relatively large in size and weight, smooth, oval-shaped and slightly flattened, covered with a white seed coat with a specific regular marble pattern. The seeds from the second group were significantly smaller or lighter, with distorted oval shape, often stained brownish or without the clear marble pattern. The above-mentioned poor morphological features, occurred separately or accumulated in one seed.

The lupine seeds in each group were measured and weighed. It turned out that the yield of the irrigated plants was of inferior quality (Figure 1e,f). Their seed size and weight were smaller by about 25% and about 30%, respectively. Because the percentage of abnormal seeds in both groups of plants was similar, i.e., 32% and 30% for the control and irrigated plants, respectively, as well as about 35%–40% of the abnormal ones did not germinate, in subsequent studies only normal seeds were taken into account.

### 2.3. DNA Content

**Figure 2:**
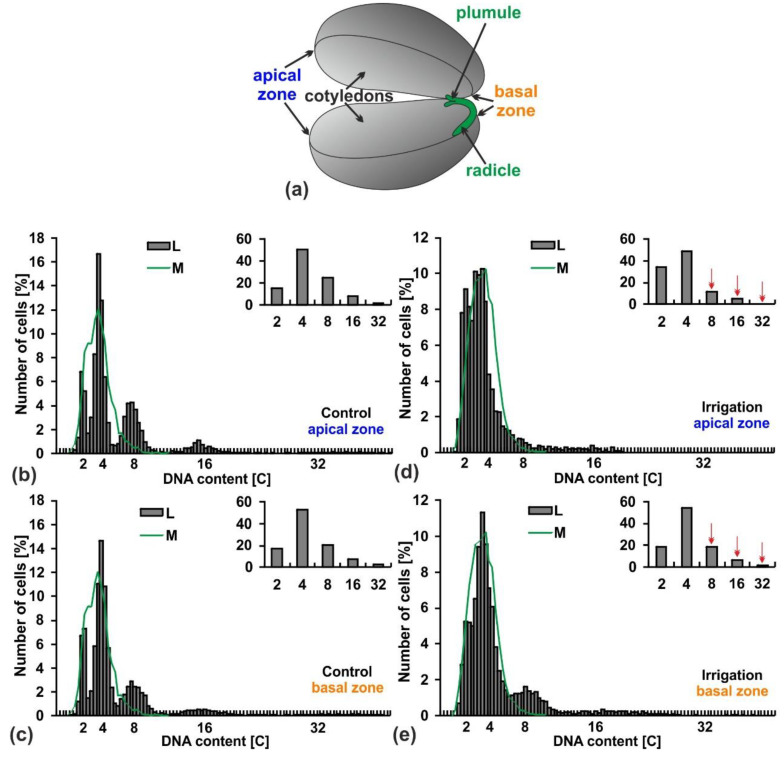
DNA content in the indicated zones of yellow lupine seeds. (**a**) Structure of lupine seed. (**b**–**e**) Frequency distribution [%] of nuclear DNA content in the selected zones: cotyledon zones (L) and root meristems (M) of yellow lupine; (**b**) Apical zone of control seeds from not irrigated plants. (**c**) Basal zone of control seeds from non-irrigated lupine plants. (**d**) Apical zone of seeds from irrigated plants. (**e**) Basal zone of seeds from irrigated plants. Inserted bar graphs show percentages of cells after successive rounds of endoreplication. Red arrows show a decrease in the number of polyploid cells in the seeds from irrigated plants.

Mature seeds of lupine have large cotyledons; their cells, depending on the area in which they are located, may be of different ploidy level and thus may occur at different sizes. Cytophotometric measurements of DNA content in the cell nuclei of two extremely situated cotyledon zones (basal and apical; Figure 2) in the control seeds did not reveal differences (Figure 2b,c). In both zones, besides the 2C and 4C DNA cells (nearly 70%), polyploid ones were also observed (about 30%). More than 20% of them passed the first round of endoreplication and contained 8C DNA, while about 7% passed two rounds of endoreplication reaching 16C DNA. A few (about 2%) contained 32C DNA.

In the irrigated seeds there was a weakly pronounced difference in the number of polyploid cells in the basal zone of cotyledons (less by only 3%), while a significantly lower number of them (less by 17%) was observed in apical zone (Figure 2d,e). In this zone, cells with 2–4C DNA content characteristic of the regular cell cycle accounted for 84% and polyploid ones for only 16% (Figure 2d). A decrease in the number of polyploid nuclei was mainly related to a significant quantitative reduction of the cells in the first round of endoreplication (Figure 2e).

### 2.4. Protein Profiles

**Figure 3:**
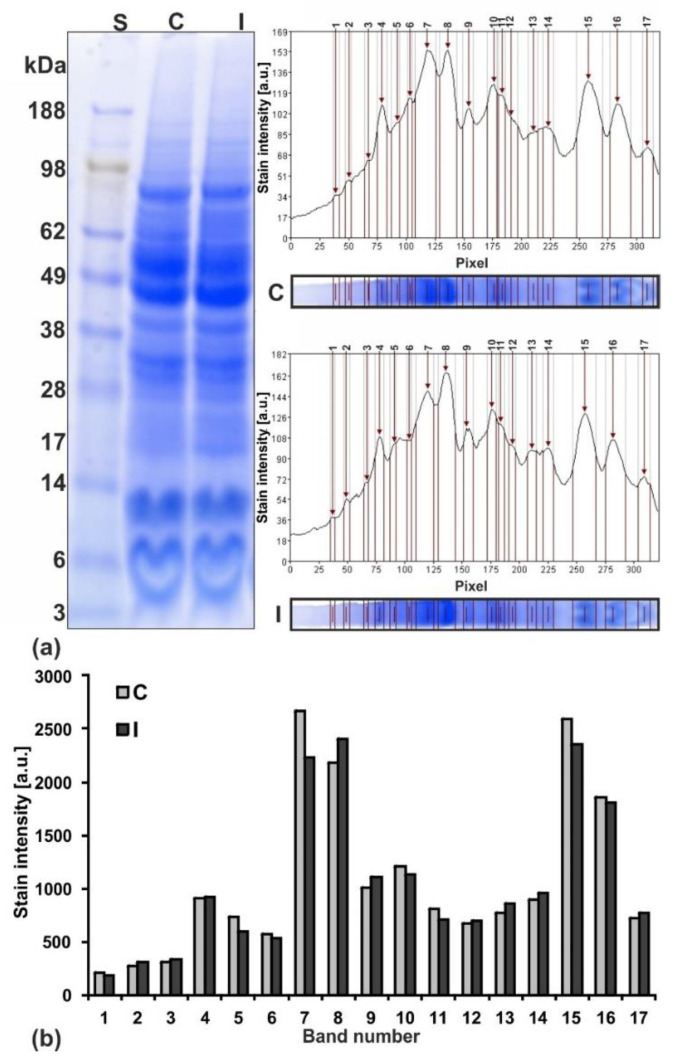
Protein profile in yellow lupine cotyledons from the seeds collected from not irrigated—(control C) and irrigated—(I) plants. (**a**) Electrophoretic separation of proteins in polyacrylamide gel (stained with Coomassie Blue) and computer analysis of staining intensity of the detected bands. Channel 1 shows protein mass standard (S), two and three show seed proteins from non-irrigated (control C) or irrigated (I) plants, respectively. (**b**) Comparison of protein contents in 17 detected bands.

Electrophoretic distribution of the proteins in the polyacrylamide gel allowed us to assess protein composition of the control and irrigated seeds (Figure 3a,b). The same number of distinguishable bands in both channels indicated the presence of similar protein composition in the tested seeds. The digital analysis of the intensity of their staining pointed to some differences in the amount of proteins present in them (Figure 3b). Even a small difference in the height of the bars (staining intensity) in each pair, e.g., band pairs 6 or 14 (Figure 3b) is clearly visible in the polyacrylamide gel (containing proteins of about 62 or 17 kDa, respectively; Figure 3a).

The most stained bands (4, 7, 8, 15, 16, 17; Figure 3b) contained subunits of storage proteins (the most abundant in cotyledones of lupine): albumins, i.e., δ-conglutin, globulins, e.g., β-conglutin (vicilin-like), and α-conglutin (legumin-like), as well as probably a non-storage protein, γ-conglutin. In comparison with control plants, the decrease in storage protein content in some bands (seven cases), and the increase in others (eight cases) indicated modifications of their proportions due to irrigation.

### 2.5. Fourier Transform–Infrared Spectroscopy (FT-IR) Analysis of Lupine Seeds

**Figure 4:**
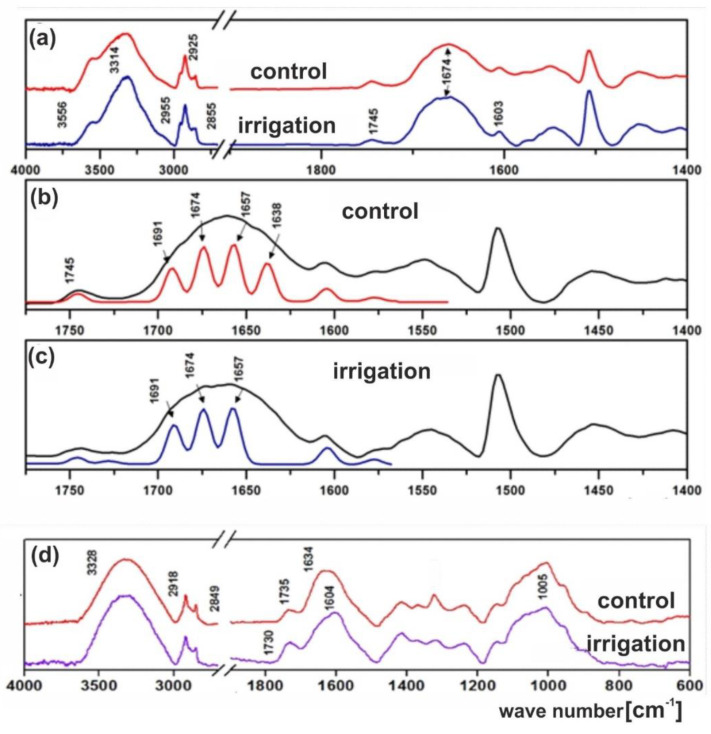
Fourier transform–infrared spectroscopy (FT-IR) spectrum of the yellow lupine seeds: (**a**) peeled seeds collected from the control (not irrigated) and irrigated lupine plants, (**b**) separation of the overlapping bands in the spectrum of the control seed—four Gaussian lines (red line) were found at 1691, 1674, 1657 and 1638 cm^−1^, (**c**) separation of the overlapping bands in the spectrum of the irrigated seed—three Gaussian lines (navy line) were found at 1691, 1674, and 1657cm^−1^. Spectra were recorded at room temperature using the diffuse reflectance infrared spectroscopy (DRIFTS) module. (**d**) attenuated total reflection (ATR)/FT-IR spectrum of the lupine seed coat cutine from the control and irrigated material. Spectra were recorded at room temperature using ATR module.

The diffuse reflectance infrared spectroscopy (DRIFTS) FT-IR spectra of dry peeled lupine seeds from control and irrigated plants are shown in Figure 4a–c. The spectrum of the every dry lupine seed exhibit two prominent absorption bands at 3314 and 1674 cm^−1^ which could be assigned to N-H and C=O stretching bands (Figure 4a). There are also three prominent bands which appear in the 2955–2855 cm^−1^ range that originate from the hydrocarbon tails. For methyl (CH_3_) and methylene (CH_2_) groups, asymmetric and symmetric C-H stretching occur at 2955, 2925 and 2855 cm^−1^, respectively. Triglyceride ester group show carbonyl C=O band at 1745 cm^−1^. The major infrared modes due to protein give rises to amide carbonyl modes in the 1700–1620 cm^−1^ range. This region consists of some overlapping carbonyl bands which may be separated using the Fourier self-deconvolution mathematical method (FSD) [36]. One of the examples of improvement of information content by using FSD method is the estimation of protein secondary structure and conformations by the analysis of the resolution-enhanced amide I profile by FSD [37].

The spectra of proteins exhibit absorption bands associated with amide groups. The exact wave numbers of C=O vibrations depend on the nature of hydrogen bonding interaction involving C=O and N-H groups. The characteristic bands of the amide groups of protein chains are similar to the absorption bands exhibited by secondary amides, and are labelled as amide I bands. It occurs between 1700 and 1600 cm^−1^.As a consequence of inter- and intramolecular interactions, the amide I bands consist of a number of overlapping component bands. The FSD-IR was used to extract individual components from a complex composite band of C=O groups. Using the deconvolution method, the ν_C=O_ characteristic stretching bands at 1691, 1674, 1657, 1638 cm^−1^ were estimated in the control material (Figure 4b). It seems most likely that changes in the composition of the seed storage proteins are due to the irrigating process (Figure 4a, navy line). There was no absorption band at 1638 cm^−1^ (Figure 4C, navy line), as compared with the spectrum of the control lupine seeds (Figure 4b, red line). We believe that the difference of wave number reflects the structural nonequivalence of carbonyl groups. It means that various protein types are present in the lupine seeds.

Figure 4d shows attenuated total reflection (ATR)/FT-IR spectra of lupine seed coats. There are four main absorption bands. The broad and intense band at 3328 cm^−1^ was assigned to the O-H stretching modes of alcohols and fatty acids. The bands in the region of 2918–2849 cm^−1^ were assigned to the stretching of aliphatic CH_2_ groups. The band at 1735 cm^−1^ was assigned to the C=O mode of carbonyl ester group. The broad band centered at 1634 cm^−1^ are due to proteins. The intense band at 1005 cm^−1^ is assigned to C–O vibration of cellulose [38].

The spectroscopic analysis demonstrated that the cuticular wax profiles of the irrigated seeds was different from the control ones. The absorption band assigned to the C=O mode of ester group was more intensive for the former ones. The broad band characteristic of proteins centered at 1634 cm^−1^ from non-irrigated seeds and at 1604 cm^−1^ from irrigated ones, as well as the broad band (characteristic of cellulose) centered at 1005 cm^−1^ also showed some differences.

### 2.6. Analysis of Chemical Elements by the SEM/Energy Dispersive Spectroscopy (EDS) Technique

**Figure 5:**
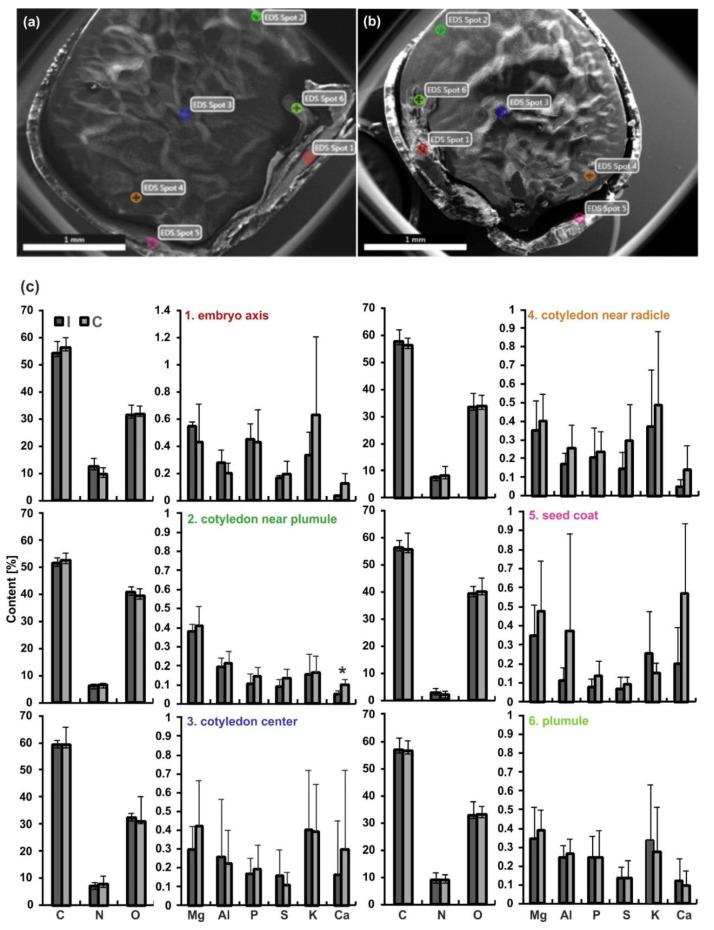
Scanning electron microscope (SEM) micrographs of yellow lupine half seeds: (**a**) Seed of a control plant. (**b**) Seed of an irrigated plant. The spots: 1-embryo axis, 2-cotyledon near plumule, 3-cotyledon center, 4-cotyledon near radicle, five-seed coat, six-plumule. (**c**) Corresponding content (weight %) of chemical elements (C, N, O, Mg, Al, P, S, K, Ca) in the indicated zones of seeds (C-control, I-irrigated plants, respectively). Statistical significance between mean values was assessed with the Student’s t-test (*p* = 0.008). Error bars represent standard deviation (SD). An asterisk indicates statistically significant results.

In the seeds of yellow lupine three chemical elements, C, O and N, were dominant. Their contents in each analyzed zone were similar in the control and irrigated seeds (Figure 5). Other elements (Mg, Al, P, S, K, Ca), whose contents (expressed in weight %) oscillated on average around 0.5% showed no statistically significant changes after irrigation treatments (Figure 5c). Among these various studied elements only the calcium content decreased statistically significantly in one seed zone (in the cotyledon near plumule), probably as a result of plant irrigation.

### 2.7. Germination and Seedlings’ Growth

**Figure 6:**
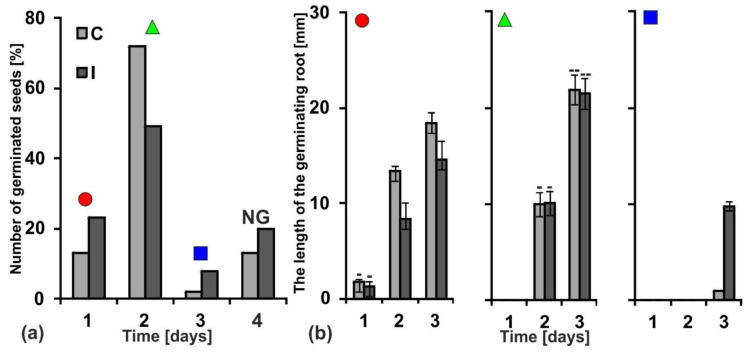
The dynamics of yellow lupine seed germination and growth of embryo roots. (**a**) Percentage of germinated seeds during three days. The seeds collected from not irrigated plants (control C). The seeds collected from irrigated plants (I). The seeds which remained non-germinated (NG) after four days. Black figures (circle, triangle, square) above bars indicate populations of germinated seeds whose root length is presented on the graphs marked with an adequate figure in part (B). (**b**) Dynamics of embryo roots growth during following days of germination. Statistical significance between mean values in diagram marked with black circle and triangle was assessed with the Mann–Whitney U test (*p* < 0.01) and the two-way ANOVA with the post-hoc unequal N HSD (honest significant difference) Tukey test (*p* < 0.01), respectively. Error bars represent standard deviation (SD). Minuses and double minuses indicate pairs of statistically insignificant results.

Substances stored in a storage tissues (for example in the cotyledons) are used during germination and growth of young seedlings. The dynamics of germination and growth are the parameters that allow to evaluate seed quality. The seeds collected from control and irrigated plants (only correct, as described in the Section 2.1., Figure 1) were germinated for three days (Figure 6). The seeds that did not sprout after three days did not sprout at all; they constituted 13% and 20% in the control and irrigated seed lots, respectively. After the first day of germination as much as 23% of the irrigated seeds sprouted out, while in the control there was almost half as much, only 13% (Figure 6a). In both groups of seeds the vast majority sprouted after two days. However, the overall percentage of germinated seeds was higher in the control (72%) than those in the irrigated material (49%).

Among the seeds that germinated after the first day, the control seedlings grew faster, whereas among those that germinated later (after two days) both control and irrigated seedlings grew similarly and finally reached larger sizes than the first ones (Figure 6b).

### 2.8. Mitotic Activity

**Figure 7:**
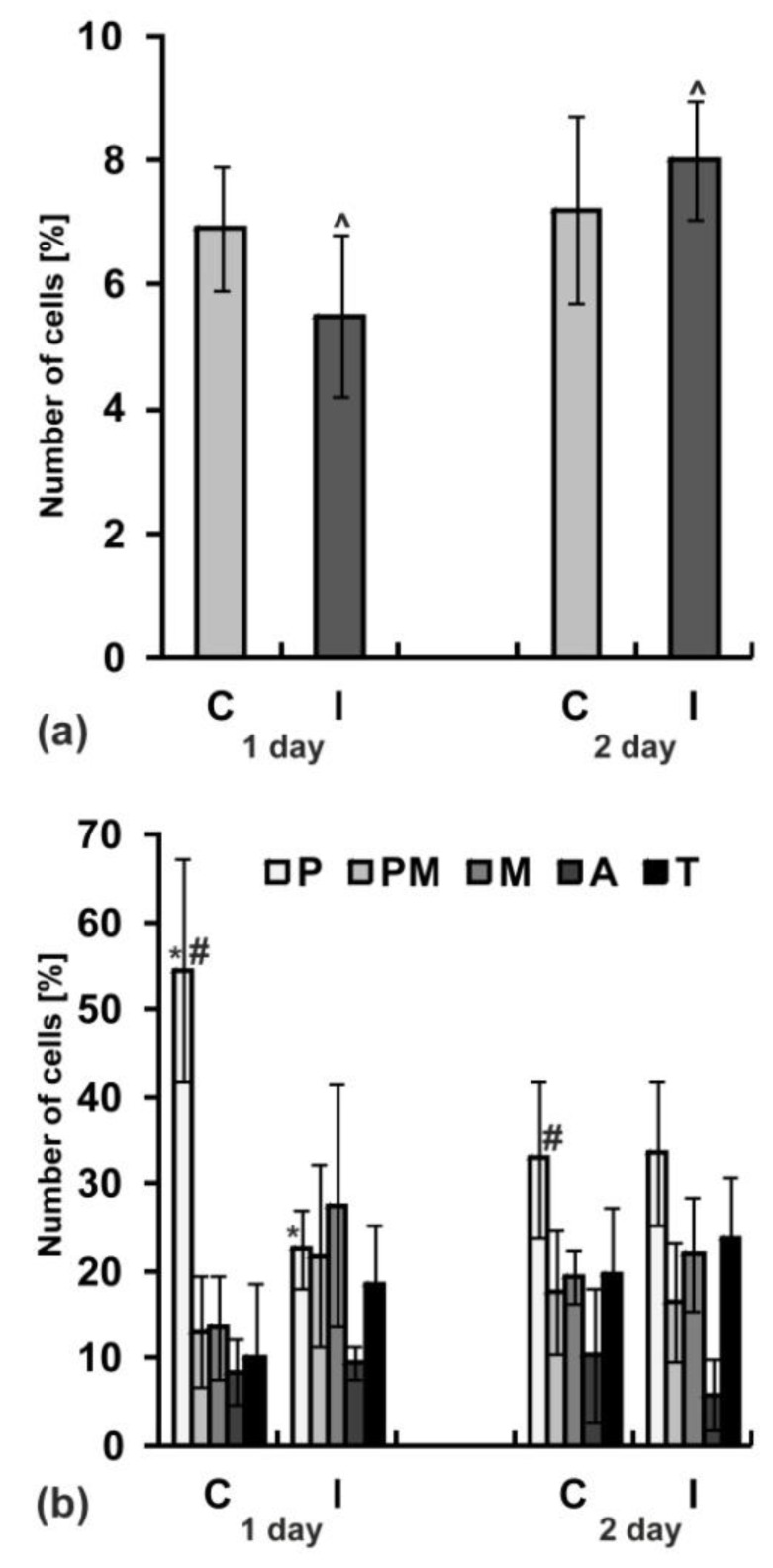
Mitotic activity in yellow lupine root meristems after one and two days of seed germination. The seeds collected from not irrigated (control—C) and irrigated—I plants. (**a**) Mitotic index, (**b**) phase index, P—prophase, PM—prometaphase, M—metaphase, A—anaphase, T—telophase. Statistical significance between mean values was assessed with the two-way ANOVA and post-hoc unequal N HSD Tukey test (*p* < 0.01). Error bars represent standard deviation (SD). Pairs of symbols (∧, ∗, #) over the bars indicate pairs of statistically significant results.

Cell proliferation in meristems is one of the main causes of seed germination and growth of the embryonic roots. Both after the first and the second day of germination, the differences between the mitotic indexes evaluated for root meristems in seeds of the control and irrigated plants were statistically not significant (Figure 7a). However, after the first day of germination in roots growing from the control seeds, cell divisions started with greater synchronization, as evidenced by the high prophase index (more than 45%; Figure 7b).

### 2.9. Detection of Hydrogen Peroxide

**Figure 8:**
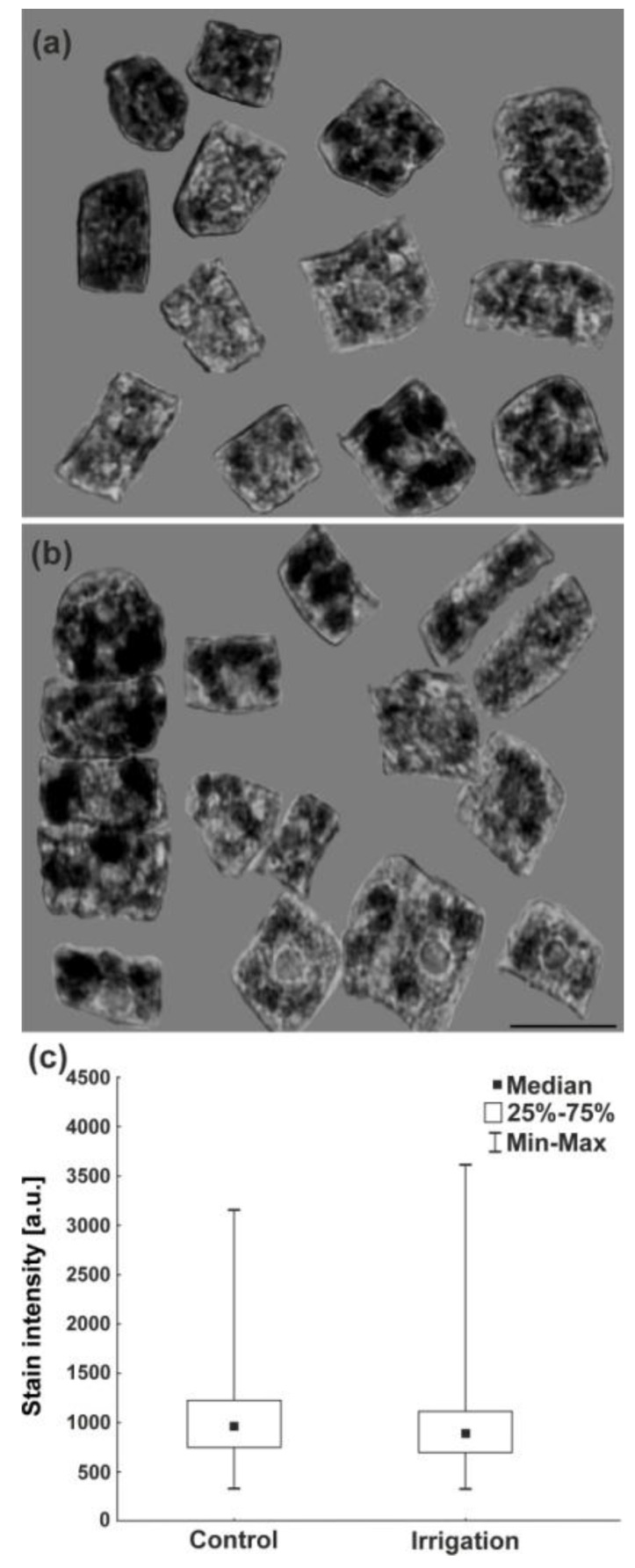
Identification of H_2_O_2_ in the form of dark 3,3-diaminobenzidine (DAB) polymers and the level of H_2_O_2_ in the cells of embryonic roots deriving from lupine seeds. (**a**) Not irrigated—control plants. (**b**) Irrigated plants. Scale bar 20 μm; (**c**) stain intensity (arbitrary units) in these cells. Statistical significance between median values was assessed with the Mann–Whitney U test (*p* < 0.01). Median values are statistically insignificant.

During germination of seeds, reactive oxygen species (e.g., hydrogen peroxide—H_2_O_2_) are produced in the embryo roots of young seedlings. Their appropriate level is necessary to promote changes in the structure of a cell wall and to facilitate elongation of cells. On the other hand, H_2_O_2_ is also a dangerous compound which adversely affects the cells. Too high a level of H_2_O_2_ causes double-strand DNA breaks and destroys the structure of chromosomes. Analyses of the H_2_O_2_ content (based on the 3,3-diaminobenzidine (DAB) polymerization method) revealed that its level was similar in the roots grown from the control and irrigated seeds (Figure 8).

## 3. Discussion

Due to the advantages of yellow lupine cultivation, treatments aimed at counteracting adverse natural conditions as well as research studies that monitor plant reactions to the prevailing and modified growing conditions are justified. In the future they will allow to increase the yield or keep it at a predictable level for years characterized by changing weather, and thus will encourage farmers to grow this species. One of the commonly used agrotechnical methods that could prevent water shortages, ensure the correct rhythm of plant development and intensify yields, is crop irrigation [5,39]. It causes an increase in the yield of cereals (up to 27%) and also improves the amount and quality of some legume seeds like chickpea beans [31,39]. However, this common agronomic operation has not been optimized for lupine seed harvest yet. Generally, lupine plants are sensitive to water deficit and intolerant of waterlogging but stress response depend on the species and plant condition. It was found that plants belonging to one genus—*Lupinus* react differently to new conditions caused by unfavorable weather occurring over the growing season [5,18,40,41,42]. Therefore, it is extremely important to optimize the growing conditions for an individual species. Surprisingly, current studies showed that irrigation of yellow lupine (*L. luteus* L. cultivar Mister) did not significantly increase yields, while it weakened the quality of seeds. Seed size was smaller by about 25% and the weight—by about 30%. However, the yield of narrow-leaved lupine cultivated at the same time and under the same irrigation conditions was even 2.5 times higher than of the non-irrigated plants, although the quality of irrigated seeds was also worse in terms of size and weight [43]. Thus, yellow lupine seems to grow better under water shortage than under the conditions limiting this stress. In turn narrow-leaved lupine showed greater tolerance of the new created cultivation conditions, as the decreases in size and weight of seeds were smaller than in the case of yellow lupine. This could be the result of the anatomical structure of narrow-leaved lupine leaves, which allowed more efficient drying of the lupine field, and thus created better conditions for seed maturation.

Seed size and weight are important physical indicators of seed quality that affects vegetative growth of the next generation of plants (e.g., seedlings’ vigour); both parameters are frequently related to the size of yield, market grade factors and harvest efficiency [44]. Generally, large seeds (e.g., of wheat, rice, oat, safflower, chickpea, sugar beet and many others species of plants) have better field performance than small seeds [44]. However, some researchers showed that cultivars of pea with lower seed mass displayed better germination than those with larger seeds [45]. Furthermore, small seeds of soybean had better germination and storage reserves utilization, as well as seedlings uniformity, which grown much faster than those from larger seeds [46]. Additionally, small seeds of safflower germinated faster and plants thereof grew higher under saline conditions [47].

To find out why yellow lupine seeds were of poor quality (mostly regarding their size and weight), the interdisciplinary research at the cellular level was carried out. To the best of our knowledge research involving microscopic, cytological, biochemical, and chemical analyses of seeds collected from the irrigated and non-irrigated yellow lupine plants has never been conducted so far.

Cytophotometric analyses of nuclei from cotyledon cells of seeds collected from the irrigated yellow lupine plants revealed lower ploidy level than those from the control plants. As demonstrated in numerous studies, polyploidization plays a key role during plant tissue and organ growth and development, both in favorable conditions and during environmental stress. A positive correlation between ploidy level and cell size, was observed in many plants, and was defined as the karyoplasmic ratio theory, which suggests that an increase in nuclear DNA content can be a driving force for cell expansion [48,49,50]. This mechanism seems to be advantageous especially when energy is limited, when rapid growth is necessary, or when terminal differentiation of some cells and their specialized functions are needed [51]. Cotyledons of lupine seeds are a reservoir of storage materials (mainly proteins) for developing embryos and growing young seedlings and should grow quickly during seed development to create space for the synthesized substances [51,52,53]. Endoreduplication associated with the production of storage materials is very common, although in some studies the correlation between endoreduplication and accumulation of storage proteins was not observed [54]. Different environmental factors can also have strong impact on the genome size [50,55,56,57,58]. Our research revealed that plant irrigation may be an inhibitory factor against switching of the classical cell cycle to the endocycles. Hence low level of ploidy in the cotyledon cell nuclei may be responsible for small seed sizes. The mechanism of this process is unknown, however it was suggested that only the ccs52 protein and protein inhibitors of cyclin-dependent kinases are of crucial importance in this case, as they inhibit cell entry into mitosis and promote endocycles [59,60]. The ploidy reduction in seeds of irrigated plants is not accidental because it was observed not only in yellow lupine but also in narrow-leaved lupine. Moreover, limitation of endoreplication, mainly in the apical zone of the seeds of both plant species, is of particular interest. This indicates the existence of a characteristic response mechanism which may be associated with the sequence of deposition of the storage compounds in specific seed areas.

Yellow lupine seeds are characterized by high protein content (44%), even higher than that in soybean (35%), white lupine (40%), and narrow-leaved lupine (34%), and thus, may be considered as a source of high quality storage proteins because of their nutritional, functional and chemical properties. Therefore preservation of the proper composition of proteins in lupine seeds during agro-technical treatments is of great importance. The lupine storage proteins are mainly globulins, which include α-, β- and γ-conglutin and their composition may differ in individual species of lupine [1,61,62,63,64]. Changes in DNA content in cotyledon cells caused by plant irrigation also encouraged us to make comparative analysis of protein profiles, because environmental stress factors may change gene expression, protein composition and their chemical structure [65,66,67]. The electrophoretic and spectroscopic (FTIR) analyses demonstrated that the seeds (cotyledons) of the non-irrigated and irrigated yellow lupine plants significantly differed with respect to their chemical composition. We believe that various protein types are present in the control and irrigated lupine seeds. However, at this stage of research, it is difficult to determine which of the observed changes are favorable or unfavorable for subsequent germination and seedling development, as well as for nutraceutical and taste properties of the seeds. This is an extremely interesting and important problem to be addressed in subsequent studies, all the more so, because the differences in the chemical composition of the seeds of irrigated and non-irrigated plants are species-specific [43].

Plant seeds are covered by seed coat and impregnated by cuticle and epicuticular waxes which protect them from environmental conditions, pathogens and insect attack [38,68,69]. This layer is also of great importance during the first stage of seed germination (imbibition). Our research revealed that irrigation of lupine plants during their cultivation affected the chemical composition of developing seeds coat. This modification influenced the subsequent germination of seeds. Similarly, as it was shown in the case of narrow-leaved lupine [43], the seeds produced by the irrigated yellow lupine plants also began to germinate faster. Due to the chemically changed coat of the seeds developed in the irrigated plants (revealed by spectroscopic analyses), the process of water absorption and seed imbibition may speed up, leading to quicker seed coat cracking and germination, similarly as it was observed in other seeds [69,70,71].

Imbibition of water causes the resumption of metabolic activity in the rehydrated seeds. During the next steps of germination catabolic enzymes (amylases, proteases) cause the breakdown of the stored substances (starch and proteins). After translocation of the hydrolyzed nutrients to the embryo proper and their subsequent assimilation, the cells of the embryo in the growing regions become metabolically very active, grow in size, begin proliferative activity and expansion to form the embryonic root and then young seedlings [72]. In order to mobilize storage substances and to make them available to the embryo axis, efficient functioning of a signaling network and activation of many genes associated with germination are necessary [73,74]. Different compounds are involved in the plant signaling network, among them sugars, hormones, nitric oxide, calcium ions (Ca^2+^), hydrogen peroxide (H_2_O_2_), and others [75,76,77,78].

Our studies demonstrated that in seeds produced by the irrigated yellow lupine plants, which began to germinate faster (like narrow-leaved lupine seeds, just after the first day), the growth of embryonic root was weaker. Probably, these seeds were not fully ready for the next phases (catabolic and/or anabolic) of germination yet, which requires adequate resources of enzymes, regulatory and signal molecules. This may also be concluded from different contents of chemical elements in the seeds, i.e., nitrogen (in the embryo axis of narrow-leaved lupine) and calcium (in the cotyledon near plumule of the yellow lupine). The appropriate level of nitrogen and suitable carbon/nitrogen balance is crucial for the gene expression during germination and young seedling growth [74], while calcium signaling is, for example, involved in the regulation of cell cycle progression and gene expression in response to abiotic stresses [75]. Since calcium content was limited in the cotyledon near plumule of the irrigated yellow lupine seeds, their embryonic roots may have grown more slowly. However, analysis of mitotic activity in the meristems of the yellow lupine embryonic roots did not show statistically significant differences between the seeds of irrigated and non-irrigated plants (which were pronounced in narrow-leafed lupine), while changes between them were observed mainly in the proportions at the first stage of mitosis. Also, no statistically significant changes in H_2_O_2_ content (clearly visible in narrow-leafed lupine) were observed. H_2_O_2_ as one of the constitutive attributes of plant root physiology together with peroxidases (Clas III, E.C.1.11.1.7.), which catalyze the reduction of H_2_O_2_ or its formation (in the peroxidative or hydroxylic cycle, respectively). These processes are connected with cell wall loosening and root elongation during seed germination [79,80,81,82]. Such a result may suggest that irrigation during the growth and development of both species of plants (in an attempt to reduce drought stress) caused modifications in slightly different branches of signaling or metabolic networks and were reflected in different responses at the cell and tissue level.

## 4. Materials and Methods

### 4.1. Plant Cultivation

The research consisted in a field experiment carried out for three consecutive years at the Złotniki Research Station *(52^◦^29′ N, 16^◦^49′ E,* Poznań University of Life Sciences, Poland). The study was conducted as a stationary experiment (in a randomized complete block design with 4 replications) on grey-brown podzolic soil (pH = 4.8 measured in 1 M KCL; 1.3% organic matter: 50–110 mg kg^−1^ P, 115–195 mg kg^−1^ K) in 4-crop rotation. The yellow lupine (*L. luteus* L., cultivar Mister, certified seeds from PHR breeder, Poznań, Poland) was sown (150 kg ha^−1^) in early April. Sowing depth was 4 cm and the row distance was 18 cm. The main plot treatments were natural rainfall (non-irrigated), and natural rainfall plus irrigations (irrigated). There was a gap of 6m in width between non-irrigated and irrigated parts of plots. Irrigations were applied during flowering, pod and seed ripening (May, June, July) when consumption of 30% of the readily available soil moisture (measured by the gravimetric method) was observed in the 0.30 m root zone. The irrigation water (of good quality, containing 114 Ca^2+^, 7.4 Mg^2+^, 0 Na^+^, 0 K^+^, <1 Fe^3+^, 356 CaCO_3_ mg⋅L^−1^; pH 7.3) was taken from a small reservoir near the experimental site. Irrigation was performed using a water pump with aluminium outlet pipes (110 mm in diameter) and a rotary sprinkler. The diameters of the nozzles were 7 mm (NAAN 233/91) and the discharge rate was 5 L⋅h^−1^ (with the operating pressure of 0.35–0.4 MPa). The main pipes with the rotary sprinkler were placed in the middle of irrigated parts of plots. The mean dose of water and time of irrigation during vegetation period were 30–35 mm and 6–7 h, respectively, while the mean daily air temperatures and total precipitation in the vegetation periods in May, June and July were 15.3, 18.4, 17.5 °C and 17.5, 62.4, 214.8 mm, respectively (data from the Agrometeorological Observatory in Złotniki).

### 4.2. Yield Assessment

Ten plants of yellow lupine were collected randomly two days before harvest and were used to measure seed yield (expressed as g per plant).

### 4.3. Seed Germination for Cytological Study

Seeds of lupine were sown on wet filter paper in Petri dishes (10 seeds/∅ 15 cm) and germinated at room temperature for maximum 4 days in the dark.

### 4.4. Cytophotometry

Apical fragments of embryo roots and cotyledons were fixed in cold Carnoy’s mixture (glacial acetic acid and absolute ethanol; 1:3; *v/v*) for 1 h. Following rehydration (70% ethanol, 30% ethanol, distilled water), the roots were hydrolyzed in 4 M HCl for 1 h and stained with Schiff’s reagent (pararosaniline; Sigma-Aldrich, St. Louis, MO, USA) according to the standard methods [83]. After rinsing in SO_2_-water and then in distilled water, fragments of cotyledons from the selected zones and 1.5-mm-long apical segments of the roots were cut off and squashed onto Super-Frost (Menzel-Gläser, Braunschweig, Germany) microscope slides. Following freezing with dry ice, cover slips were removed, and the dehydrated dry slides were embedded in Canada balsam. Nuclear DNA content was evaluated by means of microdensitometry using a Jenamed 2 microscope (Carl Zeiss, Jena, Germany) with the computer-aided Cytophotometer v1.2 (Forel, Lodz, Poland). The Feulgen-stained cell nuclei were measured at 565 nm. Microscopic slides were used also to analyze the mitotic and phase indexes.

### 4.5. Electrophoretic Separation of Proteins

P-PER Plant Protein Extraction Kit (Pierce) supplemented with Protease Inhibitor Cocktail (Sigma) was used for total protein extraction. The Lowry procedure was used to determine the total level of proteins in the solution [84]. Whole-cell protein extracts were fractionated on NuPAGE^®®^ Novex^®®^ 4–12% Bis-Tris gel, in NuPAGE^®^-MES SDS (50 mM MES, 50 mMTris, 0.1% SDS, 1 mM EDTA) buffer (pH 7.3; 200 V; 110–125 mA). Analysis of staining intensity (Coomassie™) of the bands obtained by the electrophoretic separation of proteins was carried out using the Gel Analyzer 2010a (http://www.gelanalyzer.com).

### 4.6. FTIR Analysis of Lupine Seeds

The Fourier transform infrared spectroscopy technique (FT-IR;, an analytical technique offering a possibility of chemical identification of samples) is based on the fact that chemical substances show selective absorption in infrared regions. The molecules vibrate, after absorption of IR radiations, giving rise to the spectrum of absorption [85]. The FTIR spectra were recorded in the range between 4000 and 500 cm^−1^ with a Nicolet™ 6700 spectrometer (Thermo Scientific, Waltham, MA, USA); a spectral resolution was 4 cm^−1^. The spectra were obtained using ATR and DRIFTS techniques. Room temperature reflectance spectra were recorded using a Spectra-Tech DRIFTS and ATR accessory (Spectra-Tech Inc., Hanover Park, IL, USA). Eachsample was analyzed directly on the sample cup after roughing it with silicon carbide (SiC) paper. A small disc of SiC paper was used to rub off a small amount of sample. Pieces of clean SiC paper was used as the background. For the FT-IR/horizontal attenuated total reflectance (HATR) technique, a diamond crystal was used. HATR technique provides a simple means of direct handling of plant material. The lupine samples were placed in a HATR crystal and a beam of infrared radiation is directed onto a diamond crystal. The wave of radiation extends beyond the surface of the crystal and comes into the sample. The resultant radiation was measured and plotted as a function of the wave number.

### 4.7. SEM/EDS Microanalysis

Scanning electron microscope (SEM) which produces images of samples by scanning them with a focused beam of electrons (various characteristics of the sample e.g., size and shape) was used for morphological analysis of seed samples. The EDS technique (Energy Dispersive Spectroscopy), was used to identify different chemical elements present in lupine seeds as described by He and coworkers [86] and Psaras and Manetas [87], with modifications. Mature, dry seeds of yellow lupine from control and irrigated plants (five seeds of each kind) were cut on half and without sputter coating with gold were observed with a SEM, model FEI INSPECT S50 (FEI, Hillsboro, OR, USA). X-ray microanalyses were made with the EDS system (Ametek, Weiterstadt, Germany) connected to the SEM, in six selected points of each seed (embryo axis, cotyledon near plumule, cotyledon center, cotyledon near radicle, seed coat and plumule, Figure 7a,b). In all cases the voltage was 20 kV (for micrograhs 10 kV), the pressure 60 Pa, spod size 3 and live time 30s. EDS spectra were analyzed and elements whose presence was recorded in the form of peaks summarized in tables (eZAF Smart Quant Results). The content of chemical elements (weight %) were estimated statistically.

### 4.8. Histochemical Localization of H_2_O_2_

The generation of H_2_O_2_ was observed using peroxidase-catalyzed 3,3-diaminobenzidine (DAB; Sigma) polymerization test, according to Thordal–Christensen and cowerkers [88] with some modifications [89]. Seedlings of lupine were incubated for 12 h in a solution containing 1 mg⋅mL^−1^ DAB dissolved in Tris buffer (100 mM Tris, 10 mM EDTA-2Na, 100mM NaCl, pH 7.6). Additional “negative control” series comprised of lupine seedlings incubated with 1 mM ascorbic acid (AA; Sigma). Then the roots were fixed in PBS-buffered 3.7% paraformaldehyde solution for 40 min (4 °C), washed with PBS (three times) and incubated in a citric acid buffered digestion solution (pH 5.0) containing 2.5% pectinase, 2.5% cellulose and 2.5% pectolyase, at 37 °C for 30 min. Afterwards the roots were washed with PBS and squashed onto microscope glass slides in a mixture of glycerol and PBS (9:1; v/v). H_2_O_2_ was visualized under the SMZ-2T microscope (equipped with DXM 1200 CCD camera Nikon, Tokyo, Japan) as a reddish-brown coloration.

### 4.9. Statistical Analysis

The differences between values obtained in the particular experiments were assessed with the analysis of variance (ANOVA) and following post-hoc Tukey’s test, the Student’s *t*-test or the Mann–Whitney U test. The choice of the test to the individual experiment was indicated in the description of the graphs.

## 5. Conclusions

In conclusion, our research clearly indicates that irrigation of crops in drought conditions may prevent them from drying out, but due to the lack of appropriate parameters of this agrotechnical practice, it does not always lead to higher yields. Irrigation can affect seed formation, changes the level of ploidy of cotyledon cells. Furthermore, it may interfere with the quality of storage substances and influence seed germination. In connection with the above, we believe that the studies on the modifications of stressful environmental conditions on the arable crops are necessary and justified and that the agrotechnical procedure of plant irrigation (a subject of our current work) must be carefully selected and developed for the individual plant species.

## Figures and Tables

**Table 1:** Seed yield of yellow lupine [g/plant] harvested from the main stems, branches and whole control (not irrigated) and irrigated plants.

	Main Stem	Branches	Plant
Control	3.68	0.38	4.06
Irrigation	4.14	0.94	5.08

All differences between presented pairs of mean values of seed weights are not statistically significant (the Student’s *t*-test, *p* < 0.01).

## Abbreviations

FTIR	Fourier transform infrared spectroscopy
SEM	Scanning electron microscope
EDS	Energy dispersive spectroscopy
